# The Typhoid Bacillus and Its Associates

**Published:** 1899-02-04

**Authors:** 


### THE TYPHOID BACILLUS AND ITS ASSOCIATES.

Ever since the early days of the recognition of typhoid fever as a separate disease the opinion has been strongly held by some that, notwithstanding the many proofs of its infectious nature, in some cases at least it must be capable of originating *de novo* among dwellers in filthy surroundings. Hence the term pythogenic applied to enteric fever, as indicating its origin from filth. Probably this opinion is entirely incorrect, and the researches which are now in progress, under the direction of the medical department of the Local Government Board, in regard to the conditions which promote the prolonged persistence of the typhoid organism as a saprophytic microbe growing in the soil, seem to offer a ready explanation of the occurrence of those apparently isolated cases of enteric fever on which the pythogenic theory was founded—cases, that is, which cannot be traced back to any preceding infection. The recent researches of Dr. Lorrain Smith and Dr. Tennant[1] in regard to the epidemic of typhoid fever which took place in Belfast last year are, however, of great interest, as showing that, although filth cannot itself produce enteric fever, the constant exposure to filth conditions—in other words, continuous exposure to the toxins of the bacillus coli—may have a distinct influence in rendering the system susceptible to the infection of the typhoid organism. Not only does it appear that certain strains or races of the bacillus coli are so modified by their occurrence along with the typhoid bacillus that their pathogenic power is increased, but it seems that the typhoid organism can be implanted in animals who have already been infected by the coli bacillus or their products, although before such infection they were immune to it. It has thus been possible "by the use of adjuvants," as the saying is—the adjuvant being in this case the bacillus coli—to infect animals with the bacillus typhosus which had before seemed immune to it, and the result of the investigations which have now been made has been to show that somewhat the same holds good in regard to the human body. In other words, the serum reactions indicate that in the course of typhoid fever there is also an invasion of the coli bacillus. If, then, as would appear from Sanarelli's experiments, immune animals can be made susceptible to typhoid bacillus by means of a coincident poisoning by the products of the bacillus coli, and if, as is shown by Dr. Lorrain Smith, the serum in typhoid fever gives evidence of a coincident invasion of the blood by the products of the coli bacillus, it is not difficult to believe that people who are habitually exposed to filth—that is to the coli bacillus in the wrong place—may thus render themselves peculiarly susceptible to the typhoid infection should it come their way.

[1] Brit. Med. Jour., Jan. 28.

